# Analysis of risk factors and prognostic differences in hospital-acquired thrombosis between very preterm infants and moderate to late preterm infants

**DOI:** 10.3389/fped.2026.1840027

**Published:** 2026-06-25

**Authors:** Weiwei Zhu, Yahui Zhang, Yunfeng Liu, Yan Xing

**Affiliations:** Department of Pediatrics, Peking University Third Hospital, Beijing, China

**Keywords:** catheterization, moderate to late preterm infants, prognostic, risk factors, thrombosis, very preterm infants

## Abstract

**Objective:**

Neonates have the highest incidence of thrombosis among pediatric populations, and preterm infants are at a high risk of venous thrombosis, with its risk factors remaining inconclusive and treatment criteria remaining ununified. This study aimed to explore the high-risk factors for venous thrombosis in preterm infants with varying gestational ages and catheterisation types, and to clarify the clinical characteristics, treatment strategies, and short-term prognosis of neonatal venous thrombosis, to provide evidence for clinical decision-making.

**Methods:**

A retrospective cohort study was conducted on 282 preterm infants admitted to the Neonatal Intensive Care Unit of Peking University Third Hospital from January 2014 to December 2025, including 94 cases in the thrombosis group and 188 cases in the control group. Clinical data, including maternal prenatal information, infant basic information, diagnosis and treatment course, catheter-related information, and thrombosis-related details, were collected from the electronic medical record system. SPSS 27.0 software was used for statistical analysis using the Kruskal–Wallis test, Student's t-test and Mann–Whitney U test for continuous variables; chi-square test or Fisher's exact test for categorical variables; and univariate and multivariate logistic regression models and Firth's penalised likelihood logistic regression analyses were applied to identify independent risk factors for venous thrombosis in preterm infants.

**Results:**

The incidence of venous thrombosis was 0.45% in all hospitalised neonates and 1.0% in hospitalised preterm infants, with no significant differences in baseline data such as gestational age and birth weight between the two groups (*P* > 0.05). Multivariate logistic regression analysis showed that right- and left-sided lower extremity PICC placement were independent risk factors for venous thrombosis in all preterm infants (*P* = 0.000 and 0.007, respectively). Maternal anticoagulant or antiplatelet agents use during pregnancy was a clinical predictor(*P* = 0.046). Stratified analysis by gestational age revealed that right lower extremity PICC placement(*P* = 0.000) was an independent risk factor for very preterm infants (gestational age <32 0/7 weeks), and PICC placement (*P* < 0.001) was also an independent risk factor for moderate-to-late preterm infants (32 0/7 weeks ≤ gestational age <37 0/7 weeks). Among all thrombotic groups, 96%(90/94) were associated with central venous catheterisation, including 22 cases of portal vein thrombosis (related to umbilical venous catheterisation) and 68 cases of extremity venous thrombosis (related to PICC placement). The median time from catheterisation to thrombosis was 5 (3.8, 8.0) days, with 59% were deep vein thrombosis and 59% were occlusive thrombosis. Only 34% of infants had clinical manifestations, and the detection rate of asymptomatic thrombosis increased with routine vascular ultrasound application. A total of 40% of patients received pharmacotherapy (mainly nadroparin calcium, supplemented with rt-PA), with 5% discontinuing medication due to active bleeding. For central venous catheters that were no longer in use, superficial vein thrombosis was managed by immediate catheter removal without anticoagulation. For deep vein thrombosis, anticoagulation was administered for at least 3 days, followed by catheter removal after follow-up imaging confirmed thrombus resolution or stabilisation. No thromboembolic events occurred during this process. After treatment, 98% of infants improved and were discharged; 65% of thrombi completely resolved, and 21% reduced in size before discharge, with a median resolution/reduction time of 17 (7, 31) days, and no extremity functional impairment was observed in any case. Compared with the PICC-related thrombosis group, the UVC-related thrombosis group was characterised by significantly higher birth weight (*P* = 0.010), a shorter interval between catheter placement and thrombosis (4.0 vs. 6.0, *P* = 0.019), and a shorter duration of hospitalisation (32.5 vs. 53.9, *P* = 0.003).

**Conclusion:**

Lower extremity PICC placement is a core independent risk factor for venous thrombosis in preterm infants, with maternal anticoagulant use during pregnancy perhaps being a clinical predictor for all preterm infants. Neonatal venous thrombosis is mostly catheter-related, with a high proportion of asymptomatic cases, and routine vascular ultrasound can improve its detection rate. Individualised pharmacotherapy based on clinical manifestations and thrombus characteristics is associated with a favourable short-term prognosis, and timely adjustment of an abnormal catheter position is crucial for reducing thrombosis risk. Long-term follow-up is still needed for preterm infants with venous thrombosis to monitor for late adverse outcomes.

## 1 Introduction

Neonates represent the pediatric population with the highest incidence of thrombosis, approximately 40-fold higher than that in other age groups (1). Neonatal venous thrombosis is a major cause of acute complications in neonates admitted to the neonatal intensive care unit. In the short term, it may lead to loss of vascular access, and anticoagulant therapy may increase the risk of bleeding. The morbidity of thrombotic complications can reach 12%, with a related mortality of 2% (2).

Currently, there is no consensus on the risk factors for neonatal thrombosis, especially regarding the contributions of maternal and neonatal risk factors. Moreover, there is no unified standard for the treatment of neonatal thrombosis. Clinically, management strategies—including observation alone or antithrombotic therapy—are selected based on the location of the thrombosis, whether it is occlusive, and the infant's underlying comorbidities. Although relevant clinical practice guidelines have been published, the level of evidence remains low due to the lack of randomised controlled trials in neonates (3).

This study explores thrombosis in preterm infants and further analyses the high-risk factors for thrombosis across different gestational ages and types of catheterisation, to inform clinical decision-making and clarify the therapeutic effects and short-term outcomes of interventions. These represent a future research direction (4).

## 2 Materials and methods

### 2.1 Participants and groups

This retrospective cohort study included 282 premature neonates who were admitted to the Neonatal Intensive Care Unit at Peking University Third Hospital after birth or transferred to our hospital within 24 h after birth from January 2014 to December 2025. During the study period, a total of 20,949 neonates were admitted to the Neonatology Department, among whom 9,110 were preterm infants. Patients were excluded if they died or were transferred to another hospital due to surgical diseases during hospitalisation. The preterm infants were classified into two groups (thrombosis and control) based on whether they developed venous thrombosis (*n* = 94) or did not (*n* = 188). Controls were selected from the same NICU admission pool as consecutive eligible preterm infants without thrombosis. Descriptive similarity in gestational age (±2 weeks) and birth weight (±250 g) between groups reflects the demographic characteristics of our NICU population rather than a matched design. The 32-week cutoff was chosen because it represents the clinical threshold at which our NICU typically transitions from umbilical venous catheterisation to peripherally inserted central catheter (PICC) as the primary vascular access strategy. We further stratified the cases into subgroups according to UVC-related and PICC-related thrombosis. Ethical approval for this study was granted by the Ethics Committee of Peking University Third Hospital. The ethical approval number is M20260260. Vascular ultrasound was used to confirm the diagnosis of venous thrombosis.

Maternal prenatal data, infant baseline data, diagnosis and treatment data, catheter-related data, and thrombosis-related data were extracted from electronic medical records. We collected the following data: candidate variables were pre-specified based on clinical rationale and published literature rather than statistical screening. Variables were selected *a priori* by a panel of neonatologists before data analysis.

#### 2.1.1 Maternal prenatal information

Mode of delivery, premature rupture of membranes (PROM), chorioamnionitis/antenatal fever, gestational hypertension, eclampsia or preeclampsia, gestational diabetes mellitus (GDM), autoimmune diseases, positive cervical secretion culture, prenatal administration of glucocorticoids, anticoagulants or antiplatelet agents, and antibiotics.

#### 2.1.2 Infant basic information

Gestational age, birth weight, gender, small for gestational age (SGA), twins, twin-twin transfusion syndrome (TTTS)-recipient, TTTS-donor.

#### 2.1.3 Infant diagnosis and treatment course

Neonatal asphyxia, respiratory distress syndrome (RDS), patent ductus arteriosus (PDA), persistent pulmonary hypertension of the newborn (PPHN), congenital heart disease(CHD), shock/cardiac insufficiency, sepsis, necrotizing enterocolitis (NEC), polycythemia, administration of hypertonic fluid (calcium gluconate), use of vasoactive agents, and administration of ibuprofen/paracetamol (acetaminophen). According to the 2016 Chinese Expert Consensus on the Diagnosis of Neonatal Asphyxia, severe neonatal asphyxia is defined as a 1-minute Apgar score ≤3 or a 5-minute Apgar score ≤5, combined with an umbilical arterial blood pH ＜7.0 (5).

### 2.2 Catheter-related information

Umbilical venous catheterisation (UVC), Peripherally Inserted Central Venous Catheter (PICC), postnatal age at catheter insertion, catheter tip location (central/peripheral), catheter insertion site, platelet counts before/after catheterisation, and before/after thrombosis. Vascular ultrasound protocol: Vascular ultrasound was conducted by two specialised sonographers from the hospital's ultrasound department, both with expertise in neonatal bedside critical care. A standardised scanning protocol was used, including evaluation of vessel patency, thrombus presence, extent, and echogenicity. Linear and convex probes (5–12 MHz and 5–8 MHz, respectively) were used, depending on vessel depth (Philips CX50 ultrasound systems).

#### 2.3.1 Catheter placement

UVC placement was performed by a neonatologist. PICC placement was performed by NICU nurses certified in PICC insertion. Insertion was primarily based on anatomical landmarks. Catheter tip verification: catheter tip position was verified by chest/abdominal x-ray for central placement. For UVC, the tip was targeted at the inferior vena cava-right atrial junction (at the T8-T9 vertebral level). For PICC, the tip was targeted at the superior vena cava-right atrial junction (T4-T6 vertebral level) or inferior vena cava (for lower-extremity placement,T9-T11 vertebral level). Tip position was classified as “central” (within the target vessel) or “peripheral” (extraluminal or in a non-target vessel) based on x-ray findings.

### 2.3 Thrombosis group-specific information

Time from catheter insertion to thrombosis detection, catheter removal time, clinical manifestations, treatment, and prognosis. Occlusive thrombosis was defined as the complete absence of blood flow distal to the thrombus on colour Doppler ultrasound, with vessel expansion proximal to the obstruction. Non-occlusive thrombosis was defined as partial luminal obstruction with preserved distal flow. Anticoagulant therapy was initiated for: (1) occlusive thrombosis causing clinically significant impairment of blood flow; (2) thrombosis associated with progressive symptoms (e.g., worsening limb swelling, compromised catheter function); (3) portal vein thrombosis with extension beyond the left portal vein. (4)Thrombolytic therapy was administered in cases of thromboembolism causing critical organ or limb ischemia. Expectant management (observation without anticoagulation) was chosen for non-occlusive, asymptomatic peripheral thrombosis and isolated unilateral portal vein thrombosis without extension. For catheter-related thrombosis, the catheter was immediately removed if it was non-functional, malpositioned, or no longer clinically necessary,in accordance with the 2024 ASH/ISTH guideline. For occlusive thrombosis with ongoing catheter need, anticoagulation was administered for 3–5 days before elective catheter removal when feasible, in accordance with 2012 ACCP guideline recommendations. The total duration of anticoagulation was determined dynamically based on serial vascular ultrasound findings (every 3–7 days), clinical status, and bleeding risk. Therapy was continued until thrombus resolution or stabilisation. The ultrasound follow-up schedule: follow-up ultrasounds were performed every 3–7 days during the acute phase (first 2–4 weeks) and every 1–2 weeks thereafter, with timing adjusted based on clinical status and thrombus characteristics. All infants receiving anticoagulation were monitored for bleeding complications, including daily physical examination for bruising, hematoma, or active bleeding, and serial cranial ultrasound for intracranial haemorrhage in preterm infants.

### 2.4 Statistical analysis

All statistical analyses were conducted with SPSS software (Version 27; IBM Corporation). Continuous variables were analysed using the Kruskal–Wallis test. Statistical significance was defined as a *P*-value < 0.05. For continuous data following a normal distribution, statistics were presented as mean ± standard deviation (SD);non-normally distributed continuous variables were described as median [interquartile range, IQR (Q1, Q3)]. Categorical variables were summarised as frequencies and corresponding percentages. Between-group comparisons of continuous variables were performed using the Student's t-test for normally distributed data or the Mann–Whitney U test for non-normally distributed data. For categorical variables, the chi-square (*χ*^2^) test or Fisher's exact test was utilised depending on the data characteristics. Univariate and multivariate logistic regression models were fitted to estimate odds ratios (ORs) and their 95% confidence intervals (CIs), to explore associations between explanatory variables and neonatal venous thrombosis. Given the limited sample size (24 cases in the thrombosis group with gestational age ≥32 weeks) and the presence of complete separation for some variables, we applied Firth's penalised likelihood logistic regression for multivariable analysis, which reduces small-sample bias and ensures model convergence. To evaluate the model's robustness, sensitivity analyses were conducted. For regression analyses, the unit of analysis was the individual patient. For the four infants who developed two distinct thrombotic events, only the first event was included in analyses.

## 3 Results

### 3.1 Basic information of preterm infant venous thrombosis

The incidence of preterm infant venous thrombosis among all hospitalised infants was 0.45% (94/20949). The incidence among all hospitalised preterm infants was 1.0% (94/9110). There were no significant differences in gestational age, birth weight, gender and twin status between the thrombosis group and the control group (*p* > 0.05). The data are listed in Table 1.

**Table 1:** Basic patient information.

Groups	Number (n)	Gestational Age (weeks)	Birth Weight (gram)	Male (%)	Twins(%)
Thrombosis group	94	30.2 ± 2.6	1,235 (990,1,520)	51 (54%)	38 (40%)
Non-thrombotic group	188	30.3 ± 2.6	1,225 (1,050,1,490)	81 (43%)	85 (45%)
*p* value		0.631	0.652	0.077	0.474

### 3.2 Clinical features of preterm infant venous thrombosis

Venous thrombosis occurred in 94 preterm infants. Among them, 4 infants had both portal vein thrombosis and extremity venous thrombosis; only the first event was included in analyses. Only one preterm infant had no history of central venous catheterisation after birth. Among the remaining 97 preterm infants, one infant was considered related to peripheral venous puncture. One infant was diagnosed with near-atresia of the left renal vein in the fetal period, and left renal vein thrombosis was confirmed by renal ultrasound immediately after birth; although this infant had a history of PICC placement, the thrombosis was judged to be unrelated to the catheter. One infant with portal vein thrombosis only had PICC placement but no umbilical venous catheterisation, and the thrombosis was also considered unrelated to catheterisation. The remaining 90 preterm infants had a history of central venous catheterisation, and the thrombosis was consistent with the catheterised vessel. Of the preterm infants, 22 (24.4%) developed portal vein thrombosis following umbilical venous catheterisation, while 68 (75.6%) developed extremity venous or inferior vena cava thrombosis after PICC placement. Among all cases, isolated limb venous thrombosis accounted for 75%, isolated vena cava thrombosis for 13%, and concurrent limb and vena cava thrombosis for the remaining 12%.

Umbilical venous catheters were inserted on day one after birth, and the median time to PICC insertion was 5 (4,6) days. For lower-extremity PICC placement, the great saphenous vein (92%) and small saphenous vein (8%) were used. For upper-extremity PICC placement, the basilic vein (71%), cephalic vein (14%), and median vein (14%) were used; one PICC was inserted via the jugular vein. The median time from central venous catheterisation to thrombosis detection was 5 (3.8, 8.0) days. Among all neonatal venous thrombi, deep vein thrombosis accounted for 59% (55/94), and occlusive thrombosis detected by vascular ultrasound accounted for 59% (55/94). The median time from thrombosis detection to catheter removal was 1 (0, 3.5) days.

34% (32/94) of infants presented with clinical manifestations. Before September 2024, vascular ultrasound surveillance was performed only in symptomatic infants. With the development of bedside ultrasound in the NICU, vascular ultrasound has become a routine examination before catheter removal, leading to a gradual increase in the detection rate of asymptomatic thrombosis. The proportion of asymptomatic thrombosis increased from 5% (3/62) before September 2024 to 95% (59/62) thereafter, nearly a 20-fold increase in detection rate. Clinical manifestations of extremity thrombosis mainly included swelling of the affected extremity and colour changes (redness or cyanosis), with no changes in skin temperature or dorsalis pedis artery pulse. Among all cases of portal vein thrombosis, only 1 exhibited bilateral lower extremity and lumbosacral oedema; the rest were asymptomatic. 43% (29/68) of PICC-related thrombosis were clinically manifested. All patients with vena cava thrombosis presented without clinical symptoms.Among cases of limb venous thrombosis, 49% presented with clinical symptoms.

### 3.3 Risk factors for all preterm infant venous thrombosis

#### 3.3.1 Risk factors for all preterm infants

Among all preterm infants (94 infants), compared with the control group(188 infants), the thrombosis group showed statistically significant differences in maternal eclampsia or preeclampsia during pregnancy, maternal anticoagulant use during pregnancy, neonatal asphyxia, PICC(left and right lower extremity catheter placement), PPHN, sepsis, and calcium infusion. Multivariate logistic regression analysis identified PICC in the left and right lower extremity catheter locations and maternal anticoagulant use during pregnancy as independent risk factors for neonatal thrombosis. We used the change in the clinical ultrasound protocol in September 2024 as a cutoff to adjust for potential bias. The results showed that this change in practice did not influence the identified risk factors. The data are listed in Table 2. The Hosmer-Lemeshow test showed good model fit (*P* = 0.269). All variance inflation factors (VIFs) were below 5, indicating no significant multicollinearity.

#### 3.3.2 Risk factors for very preterm infants (GA < 32 weeks)

**Table 2:** Univariate And multivariate logistic regression analyses for preterm infant venous thrombosis.

Variables	Univariate analysis		Multivariate analysis	
	Odds Ratio (95% CI)	*p* Value	Odds Ratio (95% CI)	*p* Value
Basic information				
Gestational Age (weeks)	0.976 (0.886, 1.076)	0.629	0.996 (0.832, 1.191)	0.963
Birth Weight (gram)	1.000 (0.999, 1.001)	0.798	1.001 (1.000, 1.002)	0.082
SGA	1.600 (0.833, 3.072)	0.158		
TTTS receiver	0.848 (0.214, 3.355)	0.814		
TTTS donor	2.045 (0.577, 7.247)	0.268		
Delivery(Cesarean%)	1.527 (0.768, 3.035)	0.227		
Maternal prenatal information				
PROM	0.943 (0.527, 1.688)	0.844		
Chorioamnionitis or antenatal fever	1.356 (0.468, 3.930)	0.575		
Hypertensive disorders of pregnancy	1.408 (0.856, 2.313)	0.177		
eclampsia or preeclampsia	3.078 (1.132, 8.367)	0.028*	2.061 (0.494, 8.600)	0.321
Hypothyroidism	0.420 (0.138, 1.278)	0.126		
Gestational diabetes mellitus	0.864 (0.508, 1.471)	0.591		
Autoimmune diseases	1.000 (0.389, 2.568)	1.000		
Positive cervical secretion culture	0.472 (0.171, 1.300)	0.146		
Antenatal glucocorticoids	0.803 (0.321, 2.011)	0.640		
Anticoagulants/antiplatelet agents	2.546 (1.232, 5.260)	0.012*	2.360 (1.017, 5.477)	0.046*
Antibiotics	0.629 (0.372, 1.064)	0.084		
Catheter-related information				
UVC	2.123 (0.934, 4.822)	0.072		
Correct tip position	1.074 (0.609, 1.895)	0.805		
PICC(Left upper extremity catheterization)	1.029 (0.210, 5.036)	0.972		
PICC(Right upper extremity catheterization)	2.374 (0.811, 6.950)	0.115		
PICC(Left lower extremity catheterization)	3.714 (1.583, 8.715)	0.003*	3.999 (1.466, 10.912)	0.007*
PICC(Right lower extremity catheterization)	6.857 (3.670, 12.813)	0.000*	7.961 (3.770, 16.810)	0.000*
Platelet after catheterization/before thrombosis(×10⁹/L)	0.999 (0.997, 1.001)	0.298		
Infant diagnosis and treatment				
neonatal asphyxia	2.136 (1.147, 3.978)	0.017*	1.547 (0.724, 3.303)	0.260
mild asphyxia	0.259 (0.060, 1.115)	0.070		
severe asphyxia	0.496 (0.105, 2.347)	0.376		
RDS	1.271 (0.768, 2.104)	0.350		
PDA	1.118 (0.673, 1.858)	0.667		
PPHN	2.605 (1.082, 6.274)	0.033*	1.622 (0.585, 4.499)	0.353
CHD	1.316 (0.548, 3.162)	0.539		
Hypotension/cardiac dysfunction	0.849 (0.457, 1.578)	0.605		
Sepsis	2.235 (1.195, 4.181)	0.012*	1.463 (0.699, 3.063)	0.313
NEC	1.341 (0.220, 8.164)	0.750		
polycythemia	0.663 (0.068, 6.462)	0.724		
Calcium administration	2.679 (1.020, 7.031)	0.045*	1.893 (0.614, 5.835)	0.266
Administration of ibuprofen/acetaminophen	0.536 (0.244, 1.179)	0.121		
vasoactivedrugs administration	1.570 (0.934, 2.639)	0.089		
Period (post-Sep 2024)			1.728(0.739, 4.040)	0.207

* *p* < 0.05.

Further stratification by gestational age revealed that among 70 very preterm infants (Gestational age <32 0/7 weeks), the thrombosis group differed significantly from the control group in PICC (right lower extremity catheter placement). Multivariate regression analysis demonstrated that PICC in the right lower extremity catheter location was an independent risk factor for thrombosis in extremely preterm infants. The data are listed in Table 3. The Hosmer-Lemeshow test showed good model fit (*P* = 0.526). All variance inflation factors (VIFs) were below 5, indicating no significant multicollinearity.

**Table 3:** Univariate and multivariate logistic regression analyses for very preterm infant venous thrombosis.

Risk Factors	Univariate analysis		Multivariate analysis	
	Odds Ratio (95% CI)	*p* Value	Odds Ratio (95% CI)	*p* Value
Gestational Age (weeks)			0.986 (0.793, 1.228)	0.903
Birth Weight (gram)			1.001 (0.999, 1.002)	0.380
Anticoagulants/ antiplatelet agents	2.053 (0.907, 4.647)	0.085	1.823 (0.720, 4.616)	0.206
PICC(Left lower extremity catheterization)	2.579 (0.983, 6.770)	0.054	2.687 (0.940, 7.684)	0.065
PICC(Right lower extremity catheterization)	5.288 (2.546, 10.980)	0.000*	5.482 (2.447, 12.283)	0.000*
sepsis	1.801 (0.907, 3.576)	0.093	1.464 (0.667, 3.215)	0.342
Hypothyroidism	0.155 (0.020, 1.214)	0.076		
neonatal asphyxia	1.754 (0.896, 3.431)	0.101		

* *p* < 0.05.

Among 24 moderate-to-late preterm infants (Gestational age ≥ 32 0/7 weeks and < 37 0/7 weeks), significant differences were observed between the thrombosis and control groups in maternal anticoagulant use during pregnancy, neonatal UVC placement, PICC, PDA, PPHN, sepsis, and vasoactive drug use. Multivariate Firth's penalised likelihood regression analysis showed that PICC placement was an independent risk factor for thrombosis in moderate-to-late preterm infants. Due to the small sample size, the PICC placement site was not differentiated. The data are listed in Table 4.Sensitivity analysis results are shown in Supplementary Table 1. Given the low incidence of thrombosis in preterm infants ≥32 weeks of gestation at our centre, only 24 cases were collected over a 12-year period, resulting in a limited sample size. Therefore, this part of the study is primarily exploratory. The findings from the univariate analyses suggest potential directions of association, which warrant further validation in larger, multicenter studies.

#### 3.3.3 Risk factors for moderate-to-late preterm infants (GA ≥32 and <37 weeks)

**Table 4:** Univariate and multivariate firth's penalized likelihood logistic regression analyses for moderate-to-late preterm infant venous thrombosis.

Variables	Univariate analysis		Multivariate analysis	
	Odds Ratio (95% CI)	*p* Value	Odds Ratio (95% CI)	*p* Value
PICC	16.130 (4.900, 63.370)	<0.000*	10.090 (2.890, 40.470)	<0.001*
sepsis	8.930 (1.640, 91.340)	0.010*	3.650 (0.430, 47.340)	0.241
Anticoagulants/ antiplatelet agents	5.250 (1.150, 31.280)	0.032*	4.240 (0.660, 31.110)	0.126
PPHN	5.250 (1.150, 31.280)	0.032*		
UVC	4.560 (1.520, 16.290)	0.006*		
Vasoactivedrugs administration	3.370 (1.060, 11.270)	0.040*		
PDA	2.980 (1.120, 8.290)	0.029*		

* *p* < 0.05.

Comparison of thrombosis subgroups across different gestational ages (with 32 0/7 weeks as the cutoff) revealed significant differences in antenatal glucocorticoids, antibiotic use before birth,neonatal PICC catheterisation, and RDS. The data are listed in Supplementary Table 2.

#### 3.3.4 Comparison across gestational age subgroups

Comparison between UVC-related and PICC-related thrombosis showed significant differences in birth weight,time from catheter insertion to thrombosis and length of stay. The UVC-related thrombosis group was characterised by significantly higher birth weight (1,469.1 ± 531.6 vs. 1,205.1 ± 357.5,*P* = 0.010), a shorter interval between catheter placement and thrombosis (4.0 vs. 6.0,*P* = 0.019), and a shorter duration of hospitalisation (32.5 vs. 53.9,*P* = 0.003). The data are listed in Supplementary Table 3.

### 3.4 Treatment and prognosis

Regarding treatment, 40% (38/94) of preterm infants received medical treatment. Pharmaceutical therapy was administered in 25% (6/24) of preterm infants with portal vein thrombosis and in 46% (32/69) of preterm infants with extremity or inferior vena cava, and 100%(1/1) of preterm infants with renal vein thrombosis. Pharmacological therapy was administered in 23% (5/22) of UVC-related thrombotic events and in 44% (30/68) of PICC-related thrombotic events.

The primary anticoagulant was nadroparin calcium injection, at a dosage of 100–200 IU/kg per dose, q12h. The treatment duration ranged from 2 days (minimum) to 54 days (maximum), with a median of 8 (3, 19) days.

The primary thrombolytic agent was recombinant tissue plasminogen activator (rt-PA). The initial dose was 0.1 mg/kg/h, which was gradually increased based on thrombus status, up to a maximum of 0.6 mg/kg/h. The treatment duration was 6 h to 3 days. The specific treatment course was determined based on follow-up vascular ultrasound findings.

Among them, 2 patients (5%) discontinued medication due to adverse effects (1 developed an active intracranial haemorrhage, and 1 developed adrenal haemorrhage).

After treatment,1 patient died due to critical illness,and 1 patient was transferred to a pediatric surgery hospital at 28 days of hospitalisation for suspected small intestinal stenosis,although the thrombus had resolved before transfer. The remaining 92 patients (98%) improved and were discharged.

Vascular ultrasound before discharge showed that thrombus completely resolved in 65% (61/94) of preterm infants, and thrombus reduction was observed in 21% (20/94). The median time to thrombus ultrasound-confirmed resolution or reduction was 17 (7, 31) days. These reported times reflect the interval between thrombosis detection and the follow-up ultrasound showing resolution or reduction, and therefore likely overestimate true biological resolution time. The remaining patients showed no significant change, but the infants presented without clinical manifestations, and ultrasonic examinations revealed non-occlusive thrombosis. A regular outpatient follow-up ultrasound was recommended after discharge. No patient had functional impairment of the affected extremity. There was no statistically significant difference in total hospital stay between the two groups [median [IQR]: thrombosis group 49(30,68) days vs. non-thrombosis group 45(31,67) days, *P* = 0.590].

## 4 Discussion

Our study showed that the incidence of preterm infant venous thrombosis among all hospitalised infants was 0.45% (94/20949). The incidence among all hospitalised preterm infants was 1.0% (94/9110). Our findings were consistent with those reported by Jumani et al. (incidence 0.5%) (6) and slightly higher than those by Bacciedoni et al. (2.4 per 1000 admissions) (7). Robinson et al. reported an overall thrombosis incidence of 0.20% in the NICU, with an annual incidence ranging from 0.1% to 0.25% (8). Bhat et al. conducted a retrospective case-control study and reported an incidence of neonatal venous thrombosis of 11.5 per 1,000 NICU admissions, which was markedly higher than that in the present study (9). The considerable variability in incidence across studies may be attributed to differences in study populations and the frequency of vascular ultrasound monitoring. With the advancement of bedside critical care ultrasound, our NICU has shifted from symptom-based screening to routine vascular ultrasound before catheter removal, leading to an increased detection rate of asymptomatic thrombosis. Detection bias is a key contributor to the heterogeneity of reported incidence across studies. However, further research is warranted to determine whether routine ultrasound screening should be recommended in clinical practice.

Our study demonstrated that PICC placement, particularly lower extremity insertion (predominantly via the great saphenous vein), was an independent risk factor for venous thrombosis in all preterm infants. Compared with left lower-extremity PICC placement, right lower-extremity placement was associated with an approximately 2-fold higher risk of thrombosis. Consistent with numerous previous studies, central venous catheterisation has been strongly associated with thrombosis in neonates. Specifically, studies have shown that up to 90% of neonatal thrombotic events are catheter-related, with vascular endothelial injury induced by umbilical artery/vein catheters, PICC, and indwelling catheters representing the primary pathological mechanism (10). However, few studies have examined the relationship between the catheterisation site and thrombosis. A study by van Rens et al. regarding PICC insertion practices in neonates identified the great saphenous vein as the most commonly used access vessel (11). Correspondingly, our study identified great saphenous vein catheterisation as an independent risk factor for thrombosis. Furthermore, a systematic review and meta-analysis by Chen et al. (12) confirmed that lower extremity catheterisation is an independent risk factor for PICC-related thrombosis. Interestingly, although the catheter tip position (proximal insertion) has been implicated in thromboembolic events in some studies. Dubbink-Verheij et al. (13) reported that all neonates who experienced severe PICC-related thrombotic complications underwent lower extremity PICC placement, with the catheter tip positioned at or below the L1 vertebral body level (L1 to S1). Our study failed to demonstrate such an association. No such association was identified in our study, which may be due to timely adjustment of abnormal catheter positions upon detection and to the regular ultrasound monitoring of catheter positions in our NICU. Our findings underscore the need for caution when performing lower extremity PICC,particularly on the right side. If an abnormal tip position of the indwelling catheter is identified, timely adjustment and regular monitoring are required.

Our study demonstrated that maternal anticoagulant use during pregnancy may be an independent risk factor for venous thrombosis in all preterm infants,which may be associated with maternal eclampsia or preeclampsia during pregnancy. To date, no studies have investigated the effects of prenatal administration of low-molecular-weight heparin (LMWH) and low-dose aspirin (LDA) on the coagulation function of neonates. In our study, LMWH was mainly administered to pregnant women with autoimmune diseases, including antiphospholipid syndrome and systemic lupus erythematosus, as well as those with hypercoagulable states and cerebral venous sinus thrombosis. Low-dose aspirin was primarily used for pregnant women at risk of preeclampsia. Most previous studies have focused on the effects of LMWH and LDA on mothers and fetuses, while investigations into neonatal outcomes have been largely confined to reductions in perinatal mortality and in the incidence of small-for-gestational-age infants, as well as increases in neonatal birth weight (14, 15). No studies have investigated the effects of these drugs on coagulation function in neonates. Low-molecular-weight heparin (LMWH) does not cross the placental barrier, and information pertaining to antiplatelet agents is far more limited than that for anticoagulants (16). Therefore, the authors propose that it is not the anticoagulant itself but the underlying maternal diseases necessitating anticoagulant administration that render neonates more susceptible to thrombotic events after birth. Our previous research has identified maternal autoimmune diseases during pregnancy as an independent risk factor for neonatal thrombosis (17). Maternal autoimmune diseases are the primary indication for anticoagulant therapy. Additionally, existing studies have demonstrated that preeclampsia is an independent risk factor for neonatal thrombotic events, which may lead to vascular endothelial cell injury and induce thrombosis (18). Thus, greater attention should be paid to the underlying maternal diseases(including eclampsia or preeclampsia and maternal autoimmune diseases) that indicate the use of the aforementioned medications.

Our study showed that among 24 preterm infants with portal vein thrombosis (PVT), 22 had a history of umbilical vein catheterisation (UVC), and all thromboses occurred in the left sagittal segment of the portal vein. Neonatal portal vein thrombosis (PVT) is a rare but clinically significant vascular complication often associated with the use of umbilical vein catheters (UVCs). Potential clinical manifestations of PVT include unexplained thrombocytopenia, obstruction of catheter infusion, increased pressure in the UVC line, and poor perfusion of the lower body/limbs; however, the disease may also be completely asymptomatic (19). In this study, only 1 case presented with oedema of the lower extremities and lumbosacral region, while the remaining cases had no clinical manifestations. Therefore, the diagnosis primarily relies on vascular ultrasound. Currently, there is no unified standard for the treatment of neonatal PVT. In this study, anticoagulant therapy was administered in 23% of thrombotic events. These thromboses were confirmed as occlusive thrombosis by vascular ultrasound examination according to the ASH/ISTH guidelines (2018 and 2024) (20, 21). All infants were discharged with improvement. In a previous study by Dubbink-Verheij et al., expectant management was the main approach, with a lower proportion of drug treatment (approximately 6.7%, 2/30). Among those receiving expectant management, 87% of the thromboses resolved spontaneously (22).In a study by Cabannes et al., 53 neonates with PVT underwent UVC removal without anticoagulant therapy. After 1 year of follow-up, only 5 infants (4%) still had isolated left portal vein thrombosis with no resolution (23). A study by Morag et al. showed that there was no significant correlation between anticoagulant therapy and prognosis; during a follow-up period of several years (mean 5.7 years), 27% of neonates with PVT developed adverse outcomes (30 cases of hepatic lobe atrophy and 6 cases of portal hypertension) (24).In conclusion, the prognosis of neonatal unilateral portal vein thrombosis is favourable, and expectant management can be the main strategy (25). However, long-term follow-up for several years is required to monitor for adverse outcomes (26).

Our study showed that among 69 preterm infants with extremity venous thrombosis or central venous caval thrombosis, 68 had a history of a peripherally inserted central catheter (PICC). Among all cases, isolated limb venous thrombosis accounted for 75%. All patients with vena cava thrombosis presented without clinical symptoms. Nearly half of the cases of limb venous thrombosis presented with clinical symptoms. In this study, anticoagulant therapy was administered in 44% of thrombotic events. If the catheter was nonfunctional or no longer necessary, it was removed immediately, and no serious complications, such as pulmonary embolism, occurred. If the catheter remains functional and continued venous access is required, it could be kept in place while anticoagulation therapy is administered. If symptoms progress despite anticoagulation, catheter removal is warranted. For pediatric patients with symptomatic central venous access device (CVAD)-related thrombosis who no longer require venous access or whose CVAD is nonfunctional, the ASH/ISTH guideline (2018) panel suggests delaying CVAD removal until after anticoagulation initiation (days), rather than immediate removal. Antithrombotic Therapy and Prevention of Thrombosis, 9th ed: ACCP Guidelines (2012) recommend administering anticoagulant therapy for 3 to 5 days. Chalmers E et al. suggest 2 to 4 days (27). However, the ASH/ISTH guideline (2024) recommends either immediate or delayed removal of the CVAD.In this study, all infants who had their catheter removed on the day of thrombosis were managed without anticoagulation, and no pulmonary embolism or other complications were observed, indicating that immediate removal without prior anticoagulation might be a safe option. Nevertheless, larger-scale investigations are required to validate these observations (28).

The optimal duration of anticoagulation is currently unknown. The ACCP Guidelines (2012) recommend a total anticoagulation duration of 6 weeks to 3 months for CVAD-related thrombosis. The ASH guideline (2018) panel suggests using anticoagulation for ≤3 months for provoked deep vein thrombosis(VTE). The ASH/ISTH guideline (2024) panel suggests 6 weeks of anticoagulation for select pediatric patients with provoked VTE. Girardi L et al. suggest a minimum of 3 months for CVAD-related thrombosis. With the continuous advancement of vascular ultrasound technology, based on our centre's treatment experience, it is suggested that the course of treatment be determined in combination with dynamic vascular ultrasound monitoring results. The median duration of anticoagulant therapy in our centre was 8 (3, 19) days. For portal vein thrombosis, a follow-up period of ≥5 years is recommended (29).

## 5 Limitation

This study has several limitations that should be considered when interpreting our findings:

Firstly,the routine vascular ultrasound was only implemented later in the study period (2024), while earlier detection relied mainly on symptomatic cases. This introduces substantial detection bias: earlier years had underdiagnosis, later years had increased detection of asymptomatic thrombosis. This affects the reported incidence and apparent proportion of asymptomatic cases.

Secondly, this study was a retrospective case–control study, and controls were enrolled through restricted baseline selection according to gestational age and birth weight to balance intergroup confounding. Such artificial screening may inevitably introduce mild selection bias and limit the generalizability of the results.

Thirdly, this study was a single-centre investigation with a relatively small sample size. Further multicenter studies with larger cohorts are warranted to facilitate the future development of a predictive model for neonatal thrombosis.

## 6 Conclusions

In conclusion, PICC in the left or right lower extremity is an independent risk factor for neonatal thrombosis. And the risk of thrombosis in the right lower extremity is twice as high as that in the left lower extremity. Maternal history of anticoagulant or antiplatelet agent administration during pregnancy may be a clinical predictor; attention should be paid to the impact of maternal underlying conditions, including autoimmune diseases and preeclampsia or eclampsia. Additionally, timely adjustment of the catheter tip position and regular ultrasound monitoring are required following catheterisation. Expectant management is the primary strategy for isolated unilateral portal vein thrombosis, while long-term follow-up is necessary for these infants. Regarding neonatal PICC-associated thrombosis, there is currently no consensus on whether anticoagulation should be administered prior to catheter removal or on the optimal total duration of anticoagulation therapy. Prospective studies are needed to explore these issues.

## Data Availability

The original contributions presented in the study are included in the article/Supplementary Material, further inquiries can be directed to the corresponding authors.
