# Supplementary material for: Analysis of risk factors and prognostic differences in hospital-acquired thrombosis between very preterm infants and moderate to late preterm infants
Source: Front Pediatr. 2026 Jun 25;14:1840027. doi: 10.3389/fped.2026.1840027 (PMC13345825; doi:10.3389/fped.2026.1840027)
Supplement: Supplementary file 1 [file Supplementaryfile1.docx]

Supplementary Material


**Supplementary Table 1. Sensitivity analysis: association between PICC placement and neonatal thrombosis**

| mode(moderate-to-late preterm infant venous thrombosis) | OR | 95% CI | P value |
| --- | --- | --- | --- |
| Main analysis (adjusted for sepsis + anticoagulants) | 10.09 | 2.89 - 40.47 | < 0.001 |
| Sensitivity analysis 1 (adjusted for sepsis) | 10.09 | 3.67 - 49.11 | < 0.001 |
| Sensitivity analysis 2 (adjusted for anticoagulants) | 10.09 | 4.23 - 55.98 | < 0.001 |

Note: All models were fitted using Firth penalized likelihood logistic regression.

Sensitivity analyses: To assess model robustness, two sensitivity analyses were conducted. The first included PICC placement and sepsis, while the second included PICC placement and maternal use of anticoagulant/antiplatelet agents. The results showed that PICC placement remained significantly associated with neonatal thrombosis in all sensitivity analyses (Sensitivity analysis 1: OR ≈ 10.09, 95% CI: 3.67–49.11; Sensitivity analysis 2: OR ≈ 10.09, 95% CI: 4.23–55.98). The odds ratios and significance levels were highly consistent with those of the main analysis, indicating that the findings are relatively robust.

**Supplementary** Table 2. Differences in neonatal thrombosis among different gestational age groups

|  | <32 weeks group(n=70) | ≥32weeks group (n=24) | t /χ² value | P value |
| --- | --- | --- | --- | --- |
| SGA(n%) | 18.5% | 25.0% | 0.458 | 0.559 |
| PROM(n%) | 28.6% | 8.3% | 4.083 | 0.052 |
| Chorioamnionitis or antenatal fever(n%) | 8.6% | 0% | 2.197 | 0.332 |
| Hypertensive disorders of pregnancy(n%) | 48.6% | 62.5% | 1.389 | 0.344 |
| Hypothyroidism(n%) | 1.4% | 8.3% | 2.758 | 0.159 |
| Gestational diabetes mellitus(n%) | 28.6% | 37.5% | 0.668 | 0.449 |
| Autoimmune diseases(n%) | 0% | 8.3% | 5.960 | 0.063 |
| Positive cervical secretion culture(n%) | 7.1% | 0% | 1.811 | 0.220 |
| Antenatal glucocorticoids(n%) | 79.2% | 95.7% | 6.285 | 0.024^*^ |
| Anticoagulants/antiplatelet agents(n%) | 18.6% | 20.8% | 0.059 | 0.772 |
| Antibiotics(n%) | 38.6% | 8.3% | 7.660 | 0.009^*^ |
| UVC(n%) | 94.3% | 83.3% | 2.753 | 0.196 |
| PICC(n%) | 88.6% | 62.6% | 8.200 | 0.011^*^ |
| Correct tip position(n%) | 74.3% | 70.8% | 0.109 | 0.791 |
| Left upper extremity catheterization(n%) | 5.1% | 0% | 0.795 | 1.000 |
| Right upper extremity catheterization(n%) | 8.5% | 6.7% | 0.052 | 1.000 |
| Left lower extremity catheterization(n%) | 15.3% | 20.0% | 0.198 | 0.699 |
| Right lower extremity catheterization(n%) | 71.2% | 73.3% | 0.027 | 1.000 |
| Platelet before thrombosis(×10⁹/L) | 218(168,306) | 218(148,314) | -0.601 | 0.548 |
| Platelet after thrombosis(×10⁹/L) | 367.09 ± 176.08 | 322.24 ± 112.33 | -1.100 | 0.274 |
| neonatal asphyxia(n%) | 28.6% | 16.7% | 1.332 | 0.291 |
| mild asphyxia(n%) | 22.9% | 12.5% | 1.189 | 0.382 |
| severe asphyxia(n%) | 5.7% | 4.2% | 0.085 | 1.000 |
| RDS(n%) | 71.4% | 29.2% | 13.374 | 0.000^*^ |
| PDA(n%) | 65.7% | 58.3% | 0.890 | 0.641 |
| PPHN(n%) | 10.0% | 20.8% | 1.884 | 0.176 |
| CHD(n%) | 11.4% | 4.2% | 1.089 | 0.439 |
| Hypotension/cardiac dysfunction(n%) | 17.1% | 25% | 0.713 | 0.386 |
| Sepsis(n%) | 27.1% | 20.8% | 0.374 | 0.600 |
| NEC(n%) | 1.4% | 4.2% | 0.643 | 0.447 |
| polycythemia(n%) | 1.4% | 0% | 0.347 | 1.000 |
| Calcium administration(n%) | 8.6% | 16.7% | 1.232 | 0.271 |
| Administration of ibuprofen/acetaminophen(n%) | 12.9% | 4.2% | 1.420 | 0.443 |
| vasoactivedrugs administration(n%) | 42.9% | 33.3% | 0.673 | 0.476 |

Supplementary Table 3.Differences between UVC-related and PICC-related thrombosis groups

|  | UVC group (n=22) | PICC group (n=68) | t /χ² value | P value |
| --- | --- | --- | --- | --- |
| Gestational Age (weeks) | 30.7±2.6 | 29.8±2.4 | 1.551 | 0.124 |
| Birth Weight (gram) | 1469.1±531.6 | 1205.1±357.5 | 2.651 | 0.010^*^ |
| SGA(n%) | 22.7% | 19.4% | 0.113 | 0.764 |
| Delivery(Cesarean%) | 86.4% | 85.3% | 0.015 | 1.000 |
| PROM(n%) | 13.6% | 26.5% | 1.531 | 0.260 |
| Chorioamnionitis or antenatal fever(n%) | 9.1% | 5.9% | 0.275 | 0.632 |
| Hypertensive disorders of pregnancy(n%) | 45.5% | 55.9% | 0.726 | 0.465 |
| Hypothyroidism(n%) | 4.5% | 4.4% | 0.001 | 1.000 |
| Gestational diabetes mellitus(n%) | 36.4% | 26.5% | 0.792 | 0.422 |
| Autoimmune diseases(n%) | 0% | 10.3% | 2.456 | 0.188 |
| Positive cervical secretion culture(n%) | 0% | 7.4% | 1.713 | 0.329 |
| Antenatal glucocorticoids(n%) | 86.4% | 95.6% | 2.273 | 0.154 |
| Anticoagulants/antiplatelet agents(n%) | 18.2% | 20.6% | 0.060 | 1.000 |
| Antenatal antibiotics(n%) | 22.7% | 33.8% | 0.955 | 0.430 |
| neonatal asphyxia(n%) | 22.7% | 25.0% | 0.046 | 1.000 |
| mild asphyxia(n%) | 9.1% | 22.1% | 1.824 | 0.224 |
| severe asphyxia(n%) | 13.6% | 2.9% | 3.624 | 0.092 |
| RDS(n%) | 63.6% | 60.3% | 0.078 | 0.780 |
| PDA(n%) | 77.3% | 60.3% | 2.091 | 0.202 |
| PPHN(n%) | 13.6% | 10.3% | 0.188 | 0.702 |
| CHD(n%) | 4.5% | 10.3% | 0.678 | 0.674 |
| Hypotension/cardiac dysfunction(n%) | 27.3% | 17.6% | 0.963 | 0.364 |
| Sepsis(n%) | 9.1% | 29.4% | 3.716 | 0.085 |
| NEC(n%) | 0% | 1.5% | 0.327 | 1.000 |
| polycythemia(n%) | 0% | 1.4% | 0.337 | 1.000 |
| Calcium administration(n%) | 4.5% | 11.8% | 0.963 | 0.444 |
| Administration of ibuprofen/acetaminophen(n%) | 9.1% | 7.4% | 0.070 | 1.000 |
| vasoactivedrugs administration(n%) | 27.3% | 42.6% | 1.653 | 0.228 |
| partial dissolution(n%) | 13.6% | 22.7% | 0.838 | 0.543 |
| complete dissolution(n%) | 59.1% | 69.7% | 0.840 | 0.434 |
| Correct tip position(n%) | 68.2% | 76.5% | 0.600 | 0.574 |
| Time from catheter insertion to thrombosis (days) | 4.0(2.0,5.3) | 6.0(4.0,8.0) | -2.351 | 0.019^*^ |
| Platelet before thrombosis(×10⁹/L) | 209(149,302) | 219(173,313) | -0.807 | 0.419 |
| Platelet after thrombosis(×10⁹/L) | 338.0±116.7 | 372.9±174.0 | -0.859 | 0.393 |
| Pharmacological therapy(n%) | 23% | 44% | 3.200 | 0.084 |
| Time to ultrasound-confirmed improvement(days) | 24.0(6.5,34.5) | 17.0(7.0,30.3) | -0.580 | 0.562 |
| Length of stay (days) | 32.5(25.3,57.0) | 53.0(39.0,70.8) | -2.982 | 0.003^*^ |
